# Dermatology in Student-Run Clinics in the United States: Scoping Review

**DOI:** 10.2196/59368

**Published:** 2024-12-13

**Authors:** Samir Kamat, Aneesh Agarwal, Leore Lavin, Hannah Verma, Lily Martin, Jules B Lipoff

**Affiliations:** 1 Department of Medical Education, Icahn School of Medicine at Mount Sinai, New York City, NY, United States; 2 Department of Dermatology, Lewis Katz School of Medicine, Temple University, 225 Market Street, Philadelphia, PA, 19106, United States, 1 215-482-7546

**Keywords:** dermatology, dermatologist, dermatological, volunteerism, underserved population, medical education, student-run clinic, scoping review, review, PRISMA

## Abstract

**Background:**

Student-run clinics (SRCs) for dermatology hold potential to significantly advance skin-related health equity, and a comprehensive analysis of these clinics may inform strategies for optimizing program effectiveness.

**Objective:**

We aimed to perform a scoping review of the literature about dermatology SRCs across the United States.

**Methods:**

We conducted systematic literature searches of Ovid MEDLINE, Ovid Embase, and Scopus on March 1, 2023, and June 19, 2024. No date, language, or paper-type restrictions were included in the search strategy. A total of 229 references were uploaded to Covidence for screening by 2 independent reviewers (SK and LL), and 23 full-text documents were assessed for eligibility. After an additional 8 documents were identified through a gray literature search, a total of 31 studies were included in the final analysis. Inclusion criteria were as follows: (1) studies set in an SRC, which was operationally led by medical students and could render condition-relevant treatments to patients, with dermatology care; (2) published in English; (3) within the United States; (4) included characterization of any of the following: logistics, care, patients, or design; and (5) included all study or document types, including gray literature that was not peer reviewed (eg, conference abstracts, preprints, and letters to the editor). Exclusion criteria were (1) papers not published in English and (2) those with duplicated data or that were limited in scope or not generalizable. Data were extracted qualitatively using Microsoft Excel to categorize the studies by several domains, including clinic location, demographics, services offered, and barriers to care.

**Results:**

There are at least 19 dermatology SRCs across the United States. The most common conditions encountered included atopic dermatitis; acne; fungal infections; benign nevi; psoriasis; and neoplasms, such as basal cell carcinoma, squamous cell carcinoma, and melanoma. Key facilitators for the clinics included faculty oversight, attending physician participation for biopsy histopathology, and dedicated program coordinators. Major barriers included lack of follow-up, medication nonadherence, and patient no-shows.

**Conclusions:**

Dermatology SRCs serve a diverse patient population, many of whom are underrepresented in traditional dermatology settings. This scoping review provides insights to help build stronger program foundations that better address community dermatologic health needs.

## Introduction

Student-run clinics (SRCs) are one means of expanding access and delivering health care to patients who may lack or not have full comprehensive insurance. These clinics have shown positive outcomes across various health conditions, including diabetes, hypertension, depression, health screenings, and immunizations [1]. While most clinics focus on primary care needs, many SRCs have also developed a specialty focus, such as dermatology.

Historically, SRCs have typically served 2 functions: first, the primary aim is service to patients in geographic areas that may not usually qualify for safety-net programs yet still require care; second, these clinics allow medical students—the future health care workforce—an early opportunity to deliver meaningful care under the auspices of certified health care professionals [2]. SRCs typically involve medical students running all clinic components, including the logistical operations, finances, education, pharmacy, research, procedures, student or physician coordination, and overall maintaining patient safety and quality [3]. Resident physicians and attending physicians are involved in supervising care and ultimately sign off on notes and prescriptions [2]. Fortunately, clinics have also begun to incorporate specialty services, including women’s health, mental health, otolaryngology, ophthalmology, dermatology, hepatology, musculoskeletal medicine, and general surgery, thus improving the scope of services available to these historically underserved populations [2,4-6].

Within dermatology, leaders have acknowledged the importance of volunteerism to improve access to care within the field, including within the American Academy of Dermatology [7]. In an extensive survey of graduating medical students, those pursuing dermatology were less likely to care for underserved populations, conduct public health work, or practice in underserved areas [8]. Expanded opportunities for participation in SRCs may help counter these trends and encourage dermatology-bound learners to engage with underserved groups in their future careers. Learner benefits through SRCs include enhanced clinical skills, interprofessional skills, leadership, and compassion for vulnerable patient groups [9].

Despite various single-center observational studies regarding dermatology SRCs, there remains a gap in the literature regarding the state of dermatology SRCs nationwide. For example, a 2019 nationwide sample survey of free medical clinics regarding dermatology care found that half did not respond and those who did reported limited provision of dermatology care.

Operating at the intersection of medical education, health care delivery, and social justice or activism, SRCs are well positioned to address festering dermatology issues of patient access and disparities in the US health care system [2]. Thus, comprehensively characterizing all facets of student-run dermatology clinics, including demographics, patient populations, delivery model, resources, facilitators, and barriers, is important to understanding this health care delivery model and informing future efforts. Identifying and understanding historical facilitators and barriers help shape future implementation and anticipate challenges. This scoping review parallels other specialty-specific SRC reviews previously published in women’s health and ophthalmology, focusing on dermatology [10,11].

Given the already widespread nature of SRCs, we leveraged systematic methods via a scoping review (vs a narrative review) to ensure that our review was comprehensive and exhaustive. Our scoping review objective was to broadly characterize the models of dermatology SRC delivery, epidemiology of dermatology disease, and facilitators and barriers to executing dermatology initiatives within these SRCs.

## Methods

This study was performed following the Preferred Reporting Items for Systematic Reviews and Meta-Analyses Extension for Scoping Reviews (PRISMA-ScR) guidelines [12]. Our predefined protocol was uploaded to Open Science Framework on February 28, 2023.

### Literature Search

A medical librarian (LM) performed comprehensive searches to identify studies that evaluated SRCs for dermatology care. Searches were conducted on March 1, 2023, and June 19, 2024, within the following databases: Ovid MEDLINE(R) and Epub Ahead of Print, In-Process, In-Data-Review & Other Non-Indexed Citations, and Daily and Versions (1946 to June 19, 2024); Ovid Embase Classic + Embase (1947 to June 20, 2024); and Scopus. The search strategy included all appropriate controlled vocabulary and keywords for SRCs and dermatologic care. The full search strategies are available in Multimedia Appendix 1. A gray literature search was also conducted in Google and Google Scholar on February 16, 2023, and April 24, 2023 [13,14].

The 3 search engines used were selected given their comprehensive coverage and unique strengths in indexing medical and biomedical literature. The criteria enabled consideration and characterization of dermatology SRCs in the broadest sense to ensure the completeness of the scoping review. The study duration spanned 1947‐2024, incorporating 2 independent reviewers (SK, LL) with moderation by a certified medical librarian and was limited to English language studies.

All references were uploaded into Covidence (Veritas Health Innovation), an automated software to ease reference screening and selection. The references were screened by 2 authors (LL and SK) for relevance, and subsequently 23 full texts were assessed for eligibility. Discrepancies were addressed through consensus or a third-party reviewer (JBL and AA). Eight additional documents were identified through gray literature searches, contributing to a total of 31 documents included in the analysis.

### Selection Criteria

To be selected for analysis, references had to conform to the following inclusion criteria: (1) SRC study setting, which is operationally led by medical students and can render condition-relevant treatments to patients, with dermatology care; (2) English-language papers; (3) discussion of a clinic within the United States; (4) characterized by any of the following: logistics, care, patients, or design; and (5) any study or document type, including gray literature that was not peer-reviewed (eg, conference abstracts, preprints, and letters to the editor). Conversely, exclusion criteria included (1) non-English language and (2) papers with duplicated or nongeneralizable data and limited scope.

### Data Extraction and Analysis

Data were extracted from the 31 studies using Microsoft Excel (version 365; Microsoft Corp). Domains of data included conditions treated, services offered, top procedures performed, facilitators, barriers, attending volunteers, clinic location, demographics, frequency, years running, and the number of patient encounters. Data charting was completed by 2 reviewers (LL and SK). Results were qualitatively analyzed and presented under common themes (AA, SK, and HV). We used a descriptive analysis, via charting of results, of our study findings, using a predeveloped data collection instrument founded on the authors’ experiences and a literature review [15,16]. We have detailed this instrument in Multimedia Appendix 2.

## Results

### Description of Sample

**Figure 1.**
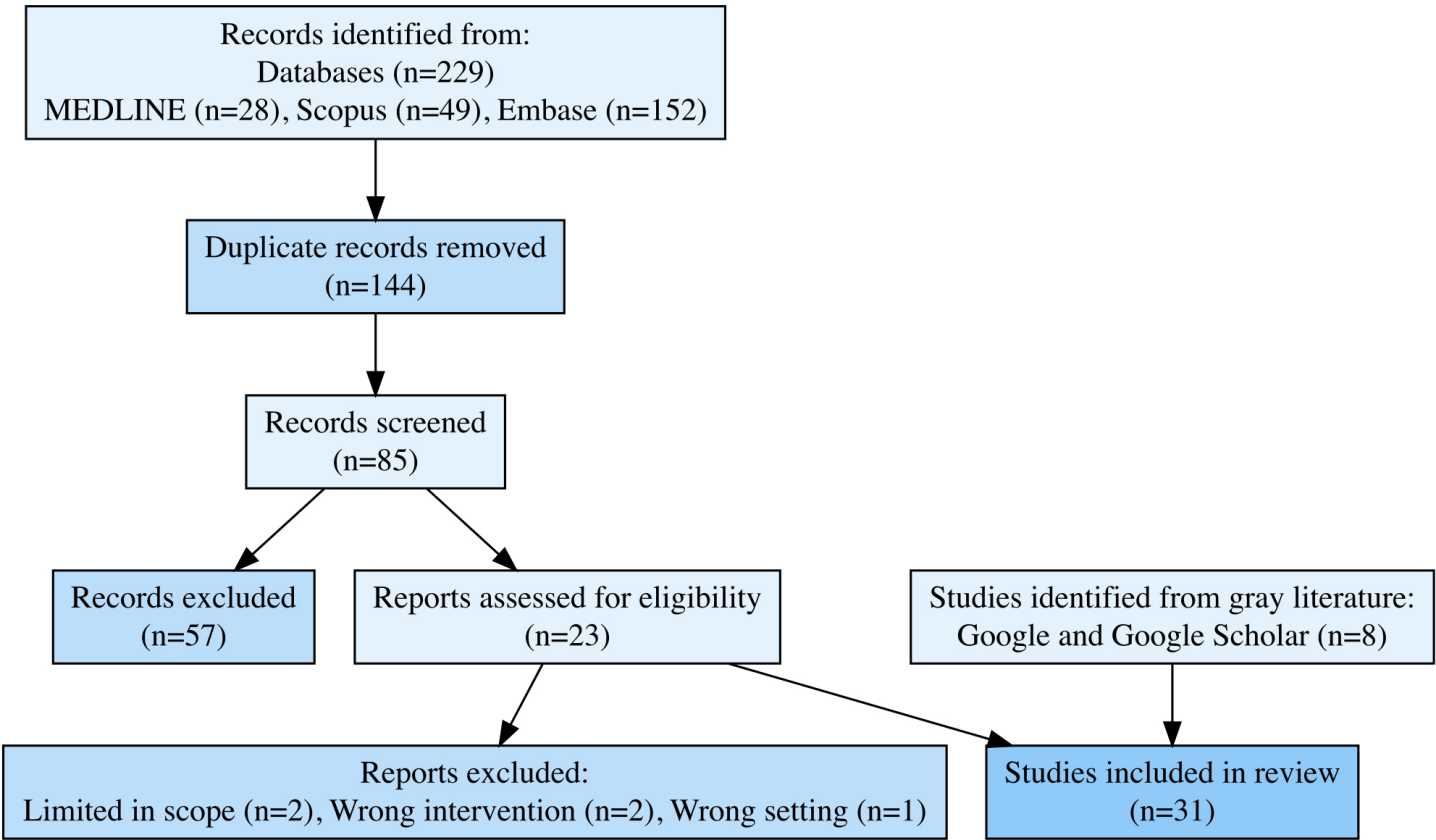
PRISMA (Preferred Reporting Items for Systematic Reviews and Meta-Analyses) flowchart of studies on dermatology in student-run clinics.

Our review included 31 studies (Figure 1) characterizing 19 student-run free clinics with a dermatology initiative in the following geographic distribution: 7 Northeast, 7 South, and 4 West (Table 1). Furthermore, we explain important aspects of dermatology within student-run initiatives through three major themes, namely: (1) patient access and prevention, (2) prominent conditions, common diagnostics, and procedural interventions, and (3) logistics and operations. The studies largely involved quality improvement projects or retrospective chart reviews. The full details of each SRCs are in Multimedia Appendix 3.

**Table 1.** Clinic characteristics.

Clinic name	Paper title	Study year	Study design	Authors	Clinic location
Free Clinic at Lubbock Impact, Dermatology Nights	Dermatologic Care for the Uninsured West Texas Population at a Student-Run Free Clinic [17]	2021	Retrospective chart review	Lin et al	Texas Tech University Health Sciences Center, Lubbock, TX
	Value of Dermatology Nights at a student-Run Free Clinic [18]	2020	Retrospective chart review	Lin et al	
HAVEN Clinic	Meeting Dermatologic Needs in an Uninsured Population: Lessons Learned From a Referrals Cohort at a Student-Run Free Clinic [19]	2021	Retrospective chart review	Mirza et al	Yale School of Medicine, Haven, CT
Teledermatology Pediatric Dermatology Clinic	Continuing Patient Care to Underserved Communities and Medical Education During the Covid-19 Pandemic Through a Teledermatology Student-Run Clinic [20]	2021	Retrospective chart review	Linggonegaro et al	Harvard Medical School & Boston Children’s Hospital, Boston, MA
UT Southwestern Student-Run Free Clinic, Dermatology Telehealth	26021 Delivering Care for the Underserved During Covid-19 Through Real-Time Teledermatology, a Cross-Sectional Review of Patients at a Student-Run Free Clinic in Dallas [21]	2021	Retrospective chart review	Rodriguez et al	Department of Dermatology, UT Southwestern Medical Center, Dallas, TX
Travis Park Dermatology Clinic	43071 Assessing the Impact of Volunteer Training at Dermatology Student-Run Free Clinic [22]	2023	Quality improvement project	Nguyen	Department of Dermatology, University of Texas Health Science Center, San Antonio, TX
	24982 Evaluation of Biopsy Management at Student-Run Free Clinic [23]	2021	Retrospective chart review	Zhu et al	
	31937 Pattern of Pediatric Skin Diseases at Student-Run Free Clinic [24]	2022	Retrospective chart review	Zhu et al	
	13093 Retrospective Review of Skin Cancer Findings at Student-Run Free Clinic [25]	2020	Retrospective chart review	Zhu et al	
	25925 Travel Burden for Free Dermatologic Care in Uninsured and Homeless Populations [26]	2021	Retrospective chart review	Patel et al	
	33929 Predominant Dermatologic Issues in Hispanic Patients at Student-Run Free Clinic [27]	2022	Retrospective chart review	Papanikolaou et al	
	Breaking Barriers: Providing Skin Cancer Education to the Homeless and Uninsured [28]	2015	Patient survey	Altshuler et al	
	25117 Analysis of Cutaneous Infections in Homeless Populations at Student-Run Free Clinic [29]	2021	Retrospective chart review	Vu et al	
	43199 Analyzing Follow-Up Rates and Barriers to Care in Student-Run Free Clinic [30]	2023	Retrospective chart review	Momin et al	
	43091 Psychodermatologic Disorders in Patient Population at Student-Run Free Clinic [31]	2023	Retrospective chart review	Nguyen et al	
	39999 Breaking Barriers in Underserved Communities and Improving Health Literacy Through a Student-Run Free Clinic [32]	2023	Quality improvement project	Zhu and Browning	
	Improving Medical Student Confidence Performing Skin Biopsies Through an Interactive Workshop [33]	2023	Pre- or posttest intervention	Nguyen et al	
Cardinal Free Clinics: Monthly Dermatology Clinic	Patient Satisfaction in Dermatologic Care Delivered by a Medical–Student-Run Free Clinic [34]	2016	Retrospective chart review/telephone survey	Pyles et al	Stanford Healthcare, Community Based-Physicians &Amp; Stanford University Student Partnership, Stanford, CA
CD Doydle Clinic (CDD)	Establishing Dermatologic Care for the Homeless and Underserved at a Student-Run Clinic [35]	2020	Quality improvement project	Teal et al	Dell Medical School, Austin, TX
HOYA Clinic	Dermatologic Education in Under-Resourced Communities: A Collaboration With a Non-Profit and a Student-Run Free Health Clinic [36]	2024	Quality improvement project	Campbell et al	Georgetown University School of Medicine, Washington DC
UCSF Student-Run Clinic at the Multi Service Center (MSC)-South Homeless Shelter	Survey of Symptomatic Dermatologic Disease in Homeless Patients at a Shelter-Based Clinic [37]	2017	Retrospective chart review	Contag et al	University of California (UCSF), San Francisco, CA
Paul Hom Asian Clinic (PHAC)	Characteristics of Patients Seen at a Dermatology Free Clinic, 2017‐2020: A Retrospective Chart Review [38]	2021	Retrospective chart review	Hai et al	University of California (UCD), Davis, CA
South Park Inn (SPI) Homeless shelter	Dermatologic Conditions in a Shelter-Based Homeless Population: Lessons Learned From a Medical Student-Run Dermatology Clinic [39]	2017	Retrospective chart review	Shahriari et al	University of Connecticut Hartford, CT
Health Outreach Partnership of EVMS Students (HOPES)	Addressing Dermatologic Health Disparities: Characterization of a Free Dermatology Clinic for an Uninsured Population [40]	2021	Retrospective chart review	O’Connell et al	Eastern Virginia School of Medicine, Norfolk, VA
Referral From Squirrel Hill Health Center, Federally Funded Community Health Center	The Student Dermatology Clinic for the Underserved: A Service-Learning Model to Promote Skin Health Equity [41]	2022	Editorial/survey	Patel et al	University of Pittsburgh Medical Center, University of Pittsburgh School of Medicine collaboration, Pittsburgh, PA
Community Health Advancement Program (CHAP)	24 Years of Student-Run Free Clinics: A Review of the Community Health Advancement Program (CHAP) Dermatology Clinic and Challenges Faced [42]	2019	Editorial	Dhami et al	University of Washington School of Medicine + Downtown Emergency Service Center (shelter), Seattle, WA
Urban Student-Run Health Clinic	Dermatological Needs in an Urban Free Health Care Setting [43]	2022	Retrospective chart review	Patel et al	University of Alabama at Birmingham Heersink School of Medicine, Birmingham, AL
Student Family Healthcare Center (SFHCC)	Assessing Skin Cancer Screening in a Student-Run Healthcare Clinic [44]	2013	Retrospective chart review	Wassef and Keller	Rutgers New Jersey Medical School, Newark, NJ
Pride Community Clinic (PCC)	40673 Evaluation of a Monkeypox Educational Intervention in a LGBTQIA+ Student Run Free Clinic [45]	2023	Quality improvement project	Alfaro et al	Department of Dermatology, University of Texas Health Science Center at San Antonio, San Antonio, TX
	41697 Predominant Dermatological Conditions in Female-to-Male Transgender Patients at Pride Community Clinic [46]	2023	Retrospective chart review	Alfaro et al	
Not identified	42662 Dermatology for the Underserved at a Non-Profit Clinic in Charleston [47]	2023	Retrospective chart review	Barker et al	Medical University of South Carolina, Charleston, SC

### Patient Demographics

#### Overview

Given the role of the dermatology SRC as a low-cost or free care option, the patient population predominantly included low-income, minority, and undomiciled individuals. A high proportion of patients were uninsured and faced language barriers [17,34]. Most clinics discussed served substantial Hispanic and Black patient populations. In studies that described housing status among patients, the rate of homelessness ranged from 44% to 100% [17,35]. The percentage of Hispanic patients ranged from 24% to 90%, exclusive of 1 Asian community clinic, that served a 78% Asian population [17,19,27,34,37-39,43]. The rate of Black patients ranged from 27% to 48% [35,37-39,43].

#### Prominent Conditions, Common Therapies, and Procedural Interventions

Common skin conditions described in the SRC population included atopic dermatitis, acne, fungal infections, benign nevi, psoriasis, and neoplasms such as basal cell carcinoma, squamous cell carcinoma, and melanoma. Among studies that calculated the prevalence of atopic dermatitis, the prevalence ranged from 10% to 49% [20,34,40]. A study that stratified diagnoses by age group found that the most common diagnosis varied by age range: atopic dermatitis being the most common in patients younger than 18 years, acne vulgaris being the most reported condition in the 18‐35 years age group, fungal infections most common in those aged 36‐49 years, xerosis most common in those aged 50‐59 years, and ichthyosis in those aged 60+ years [39]. In particular, the study describing an SRC primarily for people experiencing homelessness noted a particularly high rate of infectious conditions (74/162, 46% of diagnoses), including infestations, as well as bacterial, viral, and fungal infections [37].

Procedures included excision, shave biopsies, punch biopsies, steroid injections, and wound care [17,35,37,40,43]. In addition, topical steroids, antibiotics, and antifungals were commonly prescribed among the SRCs [29,37,40,42]. Discussion of sun protection and full-body skin checks were performed at some SRCs [25,35]. A similar spectrum of diagnoses was made via telehealth appointments at 2 teledermatology SRCs [20,21].

#### Patient Access and Prevention

A consistent issue in free SRCs is the lack of follow-up care [39,40,42,43]. Patel et al [43] found that only 57% of patients followed up with their clinic within the designated time frame, and of those who did, 19% did not adhere to their recommended medication schedule. However, incorporating telemedicine into SRC care seemed to improve follow-up attendance; 1 teledermatology clinic had a no-show rate of 9.8% (4/41) compared with the 30% no-show rate of an associated dermatology department during the same period [20]. This patient population, including homeless and uninsured individuals, faces extensive barriers to accessing care, such as language barriers, restrictive work schedules, and lack of transportation, all of which can delay or prevent follow-up. Hai et al [38] characterized the great distances their patients traveled to obtain care at the clinic, with almost two-thirds traveling more than 10 miles. At the SRC serving primarily homeless individuals, although serious conditions such as malignant neoplasms were given an immediate referral to the local hospital or private practices, follow-up was difficult for the homeless population, given the coordination required for patients’ work schedules, transportation issues, and possible misunderstandings of the health risk posed by a skin neoplasm [40].

The limited technological capabilities of patients attending SRCs also created care coordination barriers. Follow-up reminders at 1 SRC were typically sent via text or email. However, they found that undomiciled patients had unreliable access to a mobile web-based device, making it challenging to create or confirm a follow-up appointment [40]. Another SRC found that reminder phone calls before telemedicine appointments helped reduce patients’ no-show rates, although their patient population likely had more reliable access to smart devices [20].

#### Logistics and Operations

Student, resident, and attending availability was a vital issue for SRCs trying to maintain continuity of care. Issues involved seasonal availability of medical students and residents; rotating and changing supply of students, residents, and attendings; and limited number of attendings [35,39,42]. Resource limitations involving biopsy supplies were a common theme [35,37]. At 1 SRC, residents were responsible for bringing and using their own supplies and tools for excisions and treatment. However, the clinic planned to provide its own dermatology supplies, for example, liquid nitrogen, in the future [35]. At 2 SRCs, diagnostic capabilities were limited to visual inspection without histopathologic confirmation [37,39]. In addition, due to the lack of privacy inherent to the SRC based in a homeless shelter, full body skin checks and examinations were not performed, so clinicians had to rely on complaint-focused, targeted examinations [39].

Given the supplies involved in providing dermatologic care, running dermatology SRCs can incur significant costs. Using Medicare reimbursement rates for performed dermatology codes, 1 estimated value of services provided per patient ranged fromUS $61.68 to US $276.75 [18,19].

Despite these logistical and operational barriers, the student-run free clinics studied generally reported high rates of patient-reported satisfaction. Leadership and involvement of attending dermatologists were essential to several SRCs’ operations, including oversight from faculty and reliable referrals to specialists [17,19,42,43]. Attending participation was necessary for the histopathologic interpretation of biopsies taken at the free clinic, providing an essential avenue for biopsies to be read and followed through upon properly [34,41]. Another important feature of some SRCs was the incorporation of a dedicated, nontrainee program coordinator who maintained a formal infrastructure and arranged participation from attendings, residents, and medical students [41,42].

## Discussion

### Principal Findings

Our review broadly aggregates the experiences of student-run dermatology clinics across the United States. In particular, we characterized dermatology SRCs across several domains, including operational, diagnostic, treatment logistics, and overall facilitators and barriers to successful clinic function (Table 2).

**Table 2.** Overview of dermatology student-run clinics.

Domains	Conditions
Patient access and prevention	o Teledermatologyo Sun protection educationo Patient-specific barriers (language barriers, restrictive work schedules, lack of transportation)
Procedural interventions	o Excisionso Shave biopsieso Punch biopsieso Steroid Injectionso Wound care
Common therapies	o Topical Steroidso Antibioticso Antifungals
Logistics and operations	o Attending and resident availabilityo Resource limitations (biopsies, pathology confirmation) and variable costso Full-body skin checkso Complaint-focused, targeted exams

The variety of conditions encountered in SRCs is broad, similar to that seen in conventional clinics, spanning both acute and chronic dermatoses. The extent of coverage and diagnostic capability at dermatology SRCs is heavily dependent on the availability of physical, financial, and staffing resources. Frequent need for biopsy is a unique challenge to dermatology, as opposed to other specialties with SRCs [48]. Financial and logistic barriers to care remain a significant issue for dermatology SRCs in terms of capability for diagnosis and follow-up. Given the variability in financial data between SRCs and varying procedural and diagnostic services offered at each clinic, operational or financial efficacy comparisons could not be made. In addition, the up-front investment required for providing different dermatologic services imposed restrictions or limited services offered. The ability to make referrals for additional work-up or treatment of malignancy was noted as a challenge among many of the SRCs, and reliable access to dermatology attending physicians was important to ensuring high-quality care [19,20,35,41-43].

Overall, our review captures the state of dermatology SRCs across various regions and patient populations and clarifies the areas for improvement for further iteration, expansion, and creation of future SRCs. Dermatology student-run free clinics help reduce health care disparities while also training future generations of dermatologists in a manner that exposes them to diverse patient populations with vastly variable resources.

### The Potential Reach of Dermatology SRCs

The lack of access to dermatologic care for patients who are minorities, uninsured, and low-income has been well documented in the literature. In October 2022, the American Dermatological Association proposed measures to address the downstream inequities for patients with skin disease arising from unequal access, including opportunities for trainees in underserved areas [49]. SRCs, with a 2014 nationwide census of 140,000 patients and support among 75% of accredited medical schools, may help close the gap in patient access [2]. Through SRCs, medical students gain immersive exposure to social determinants of health, including health literacy and language barriers, while developing their diagnostic skills [2]. While all patients with a dermatologic complaint should eventually be evaluated by a board-certified dermatologist, due to inequities in access, initial evaluation from a medical student can help patients eventually receive a consultation from a dermatologist [50].

Robust dermatology SRCs must work in tandem with efforts to address these systemic contributors to inequity. SRCs are uniquely poised to provide free or low-cost services to patients who otherwise would not be able to access dermatology care in a timely or affordable fashion due to having Medicaid or lack of ability to pay a consultation fee [51,52]. Research has shown that Hispanic and Black patients are less likely to present at outpatient dermatologic centers [53]. Improved access to dermatologic care through SRCs may help reduce these disparities.

### Funding and Ongoing Education

Funding for dermatology diagnostic and therapeutic resources remains essential for these clinics. Our review highlighted that funding for existing efforts commonly draws from sponsoring departments and private donations. A 2007 survey of SRCs revealed private grants as familiar funding sources (71%), with a median operating budget of US $12,000 [54]. Private grants may reflect an untapped source for additional support. Pending additional resources, principles of high-value care, and quality improvement can help clinics achieve their mission with the little resources they may have. For example, one study rolled out 16 interventions over 2 months, demonstrating improvement across clinical operations and patient wait times [55]. Interventions around drug use and costs may be particularly relevant to dermatology; for example, closed formularies at 1 SRC demonstrated sizable savings while retaining similar levels of medical care [56].

Ongoing education can help ensure that care delivery evolves to meet the SRC patients’ needs. Numerous studies have identified learner’s difficulty in diagnosing conditions among skin-of-color patient populations, stemming from insufficient coverage in medical education and materials [57,58]. Fortunately, studies have additionally determined that relatively minor curriculum adjustments can help address these gaps, both for familiar and less common skin-of-color conditions, such as topical corticosteroid side effects and melanoma [59,60]. Furthermore, ongoing training and education through service learning at an SRC presents a robust means for education, having a benefits-to-cost ratio of 8.13 of clinic education expenses versus university-generated education [61]. Converting existing literature (eg, literature reviews) on the dermatology conditions around the pertinent SRC patient population into educational modules may be one way to help provide better patient show rates, context, and cultural sensitivity.

### Sustainability and Policy

Integration into a local health care system can ultimately facilitate long-term sustainability and patient continuity. A dermatology SRC is well suited as a specialty addendum for a well-established SRC already operational in the academic medical center. On the other hand, stand-alone dermatology SRCs may require greater effort to implement, over a longer term duration, and should include ongoing discussions with departments of dermatology and medical education. An initiative assessment (demonstrated in Textbox 1) may assist those interested in creating a dermatology SRC at their institution.

Textbox 1. **Sample dermatology student-run clinic initiative assessment.**

1. Does your institution have an existing student-run health care clinic?
2. What are the demographics of the target community population? What are the barriers to individuals seeking care (eg, *rural, undomiciled, immigrant,* and *uninsured*)?
3. Which and how many attendings or residents can be involved?
4. What funding and resources can be deployed (eg, *budget; availability of dermatologic supplies for therapies, biopsies, and histopathologic evaluation*)?
5. What are the ethical implications of setting up a dermatology student-run clinic in your setting (eg, *how will you ensure follow-up, sustainability*)?
6. What other facilitators and barriers to implementing a dermatology student-run clinic exist at your institution?

Teledermatology at SRCs may be a promising tool to improve flexibility for consulting attending physicians, allowing clinics to expand their reach to patients who may lack transportation. One study in Philadelphia of the community health clinic Puentes de Salud identified teledermatology as a helpful triage tool [62]. Likewise, a survey of 9499 consults in the Los Angeles County Department of Health Services also proved the triage use of teledermatology at scale across a sizeable safety-net health system [63]. One of the SRCs studied showed a sizable reduction in no-show rates for telemedicine appointments compared with in-person visits [20]. These promising findings bode well for SRCs, which have proven capable of incorporating telehealth; telehealth in SRCs has handled acute and chronic health conditions, including opioid use disorder [64-66].

Beyond SRCs, addressing the overarching issues of limited dermatologic health care access among underserved populations remains paramount. Residency-level proposals at the intersection of dermatology education and health disparities have included using existing programing across national dermatology associations and societies, implementing residency class learning projects, and collaborating with the existing community or federally funded clinics (eg, via SRCs) [67]. Broader policy interventions to expand and augment insurance coverage of underserved populations, expanded teledermatology, and regulatory flexibility are examples of relevant policy reform. Research opportunities relevant to dermatology SRCs remain plentiful and can relate to medical education, advocacy, and medical care provision. Specific examples include integrating dermatologic surgery, incorporating biologics and new dermatologic therapeutics, controlling costs, and providing a continuous supply of resources and faculty.

### Limitations

Our review has several limitations. One is that the studies in our review vary in their coverage of care characterization. Thus, not all barriers or facilitators of implementation faced by each SRC have been delineated, and comparisons between SRCs are difficult to assess. Furthermore, SRCs differ in nature depending on the affiliated institution and community resources. Thus, the findings are not generalizable to all dermatology SRC settings. In addition, our primary focus on SRCs may underreport the important role that other free or low-income clinics, such as the Puentes de Salud dermatology clinic, which involved students but was primarily run by attendings and residents, have in promoting health equity in dermatology care. Finally, while this review followed a rigorous search protocol, the search may have missed certain dermatology SRCs because they have yet to be described in the literature.

### Conclusions

SRCs have long been integral to undergraduate medical education, fostering compassion, cultural sensitivity, and a commitment to volunteerism among future physicians, while focusing on underserved populations. At the same time, dermatology has recognized the need to address health disparities and gaps in care for these communities. Dermatology SRCs contribute to these efforts by offering medical students valuable experiences at the intersection of education and community health. Despite the existence of more than 140 dermatology residency programs in the United States, we identified only 19 institutions with such clinics, highlighting significant opportunities for growth. Our scoping review provides a comprehensive overview of these clinics nationwide, with the hope of encouraging medical students, schools, and dermatology departments to establish and expand such clinics in their own communities.

## Supplementary material

10.2196/59368 Multimedia Appendix 1 Search strategy.

10.2196/59368 Multimedia Appendix 2 Data collection instrument.

10.2196/59368 Multimedia Appendix 3 Information of dermatology student-run clinics.

10.2196/59368 Checklist 1 PRISMA-ScR (Preferred Reporting Items for Systematic Reviews and Meta-Analyses Extension for Scoping Reviews) checklist.

## Abbreviations

SRC: student-run clinic
