# Supplementary material for: Dermatology in Student-Run Clinics in the United States: Scoping Review
Source: JMIR Dermatol. 2024 Dec 13;7:e59368. doi: 10.2196/59368 (PMC11661691; doi:10.2196/59368)
Supplement: Multimedia Appendix 1 [file derma-v7-e59368-s001.docx]

Ovid MEDLINE(R) and Epub Ahead of Print, In-Process, In-Data-Review & Other Non-Indexed Citations, Daily and Versions <1946 to June 19, 2024>

1 Student Run Clinic/ 186

2 (student run clinic* or student led clinic* or student led free clinic* or student run free clinic* or student clinic* or SFMCP or Student-Run Free Medical Clinic* or Student-Led Free Medical Clinic*).mp. 1238

3 Dermatology/ 21825

4 exp Skin Diseases/ 1261832

5 (derm* or skin or telederm* or tele-derm*).mp. 1141728

6 1 or 2 1238

7 3 or 4 or 5 1917394

8 6 and 7 28

Embase Classic+Embase <1947 to 2024 June 20>

1 student-run clinic/ 347

2 (student run clinic* or student led clinic* or student led free clinic* or student run free clinic* or student clinic* or SFMCP or Student-Run Free Medical Clinic* or Student-Led Free Medical Clinic*).mp. 1529

3 exp dermatology/ 58814

4 exp skin disease/ 2081157

5 (derm* or skin or telederm* or tele-derm*).mp. 1755323

6 1 or 2 1529

7 3 or 4 or 5 2860293

8 6 and 7 53

Scopus (49 results on June 21, 2024)

( TITLE-ABS-KEY ( "student run clinic*" OR "student led clinic*" OR "student led free clinic*" OR "student run free clinic*" OR "student clinic*" OR sfmcp OR "Student-Run Free Medical Clinic*" OR "Student-Led Free Medical Clinic*" ) AND TITLE-ABS-KEY ( ( derm* OR skin OR telederm* OR tele-derm ) ) )
