# Supplementary material for: Dermatology in Student-Run Clinics in the United States: Scoping Review
Source: JMIR Dermatol. 2024 Dec 13;7:e59368. doi: 10.2196/59368 (PMC11661691; doi:10.2196/59368)
Supplement: Multimedia Appendix 2 [file derma-v7-e59368-s002.docx]

**Data Collection Instrument:**

1. Authors
2. Date of Study Title
3. DOI
4. Clinic Name
5. Population served
6. Clinic Location
7. Institution
8. Years Running
9. # Patient Encounters Demographics of Clinic
10. Top Conditions Treated
11. Top Procedures Performed
12. Facilitators to Starting Clinic
13. Additional barriers / boons to clinic
14. Frequency of Clinics (monthly, weekly)
15. Level of Supervision Attending Volunteers
16. What services are offered (can you do a biopsy, telemedicine)
17. Financials
