# Supplementary material for: Dermatology in Student-Run Clinics in the United States: Scoping Review
Source: JMIR Dermatol. 2024 Dec 13;7:e59368. doi: 10.2196/59368 (PMC11661691; doi:10.2196/59368)
Supplement: Multimedia Appendix 3 [file derma-v7-e59368-s003.docx]

| **Clinic Name** | **Article Title** | **Clinic Location** | **Demographics** | **Clinic Frequency & Years Running** | **# Patient Encounters** | **Most Common Condition** | **Services Offered** | **Clinic Comments** |
| --- | --- | --- | --- | --- | --- | --- | --- | --- |
| **Free Clinic at Lubbock Impact, Dermatology Nights** | Dermatologic Care for the Uninsured West Texas Population at a Student-run Free Clinic[1] | Texas Tech University Health Sciences Center, Lubbock, Texas | Uninsured patients ages 19–64 years.  Approximately 44% of the patient population is Hispanic, 55% unemployed, and 45% homeless | Monthly. Began 2011. General medicine clinic began 2009. | 5-6 patients/ specialty night | Benign skin lesions (e.g., seborrheic keratoses, benign nevi, skin tags, lipomas), NMSCs, actinic keratoses, eczematous rashes, fungal infections, psoriasis, acne, and ulcers/wounds | Various procedures: shave biopsies, ED&C, skin excisions, wound care, help getting chemotherapy drugs  ED&Cs, excision, shave removal, wound care | Main clinic pre-existed. A champion dermatologist who trained at TTUHSC spearheaded the dermatology clinic.  Rooms equipped with proper supplies including tools for shave and punch biopsies, scalpels, and suture, a hyfrecator, local anesthetics, topical hemostatic agents, and wound care materials  3 active volunteer dermatologists, pathology trained |
|  | Value of Dermatology Nights at a student-run free clinic[2] |  |  |  | 5,400 unique patients, over 8,000 patient visits.  25 to 30 patients / week. |  |  |  |
| **HAVEN Clinic** | Meeting dermatologic needs in an uninsured population: Lessons learned from a referrals cohort at a student-run free clinic[3] | Yale School of Medicine, Haven, CT | Male (72.4%), Hispanic (89.7%), Spanish-speaking, average age 40 | As needed by referral, began 2012 | 29 patients | 65.5% of referrals were rule-out malignancy. 73.9% benign lesions (top: melanocytic nevus (26.1%), seborrheic keratosis (13%, acrochordon (8.7%), keloid (8.7%)),21.7% were inflammatory conditions (acne/eczema/intertrigo/morphea/vitiligo), 4.3% malignant (all SCC) | Cancer rule-outs primarily  Among most referrals to r/o malignancy, diagnostic biopsy 75% of cases, pathology 93.8% showing as benign, single instance squamous cell carcinoma | Time to first dermatology visit 54.4 days, with 90.2% attendance; long-term management is via the student-run clinic, specialists only used as needed  Latency between the patient initially experiencing a dermatologic concern and seeking care at HAVEN was 3.2 years.   Cost average: $276.75/patient, $151.55 per visit, $795.65 total cost per year  Physicians recruited Yale School of Medicine faculty, members of the Yale Medical Group, or colleagues of current HAVEN attendings. Faculty at the Yale School of Medicine Department of Dermatology who volunteer their time and resources to care for referred HAVEN patients see them at their respective clinics. |
| **Teledermatology Pediatric Dermatology Clinic** | Continuing patient care to underserved communities and medical education during the covid-19 pandemic through a teledermatology student-run clinic[4] | Harvard Medical School & Boston Children's Hospital, Boston, MA, USA | 43.2% (age 0-5), 7 (18.9%) 6-10, 14 (37.8%) 11+; male: 43.2 % (16), female (56.8%) 21, race/ethnicity: Hispanic (40.5%) 15, black (27%) 10, Asian 10.8% (3) | Monthly, began July 2020 | 37 encounters | other (acanthos nigricans, coommon acquired nevi, ganglion cyst, kuncle pad, lipoma, pyogenic granuloma, telogen effluvium), other inflammatory conditions (friction dermatitis, irritant diaper dermatitis, juvenile plantat dermatosis, keratosis pilaris, piyrosporum folliculitis), tie between atopic dermatitis, contact dermatitis (13%) | Pandemic switched model to teledermatology | Call to confirm appointment day before - helped reduce no-show rate. Low no-show rate of 9.8% |
| **UT Southwestern Student-run free clinic, dermatology telehealth** | Delivering care for the underserved during COVID-19 through real-time teledermatology, a cross-sectional review of patients at a student-run free clinic in Dallas[5] | Department of Dermatology, UT Southwestern Medical Center, Dallas, TX | Not reported. | Not reported | 117 telehealth appointments, 134 patient complaints (over 10-week study period during COVID-19, 2020-2021) | Unspecified rashes/dermatitis (31%), neoplasms (26%), pigmentary disorders (18%), acneiform disorders (17%), and other (8%). |  | Of those 117, 67 completed their teledermatology encounter for a teledermatology show rate of 57%. |
| **Travis Park Dermatology Clinic** | Pattern of pediatric skin diseases at student-run free clinic [6] | Department of Dermatology, University of Texas Health Science Center, San Antonio, TX | 57 pediatrics patients: 57.9% (n=33) female; 42.1% (n=24) male. Age 1 month old to 17 years old; the median age 15 years | 2x/month, began 2009 | 57 children (over 3 years) | Atopic dermatitis (21.1%, n=12), acne (15.7%, n=9). Molluscum contagiosum (n=4), fungal infection (n=3), and pigmented nevi (n=3). Local treatment was prescribed in most patients (82.5%, n=47). | Not reported. | Sun protection was discussed with only 24.6% of patients (n ¼ 14).  Homeless, indigent & uninsured |
|  | Retrospective review of skin cancer findings at student-run free clinic [7] |  | Not reported. |  | 1126 dermatology pt records over 15-month time frame | 8 patients with melanoma, 10 patients basal cell carcinoma (BCC), and 3 patients squamous cell carcinoma. Clinic newly diagnosed skin cancer in several patients: 1 melanoma, 6 BCC, and 1 SCC.  Biopsy to patient communication time, 11% of patients presenting to clinic are diagnosed with cancerous / precancerous lesions | Punch and shave biopsies | QI project involving action plan for handouts with graphics, additional volunteer training, and renovations to clinic. 311 Patient surveys with improvement in comprehension of medical conditions and comprehension of treatment plans  Skin checks, biopsies, identification of malignant skin lesions, skin cancer education, serves pediatric patients |
|  | Travel burden for free dermatologic care in uninsured and homeless populations[8] |  | Over 80% within a 25-mile radius of the clinic |  | 570 (2018-2020) | Not reported. | Not reported. | Not reported. |
|  | Predominant dermatologic issues in Hispanic patients at student-run free clinic[9] |  | 43/243 reported as Spanish-speaking patients |  | 243 | Inflammatory, benign, hyperpigmentation, psoriasis, allergic origin, infectious, hair related. Benign skin conditions were the most prevalent within the Hispanic population. | Not reported. | Not reported. |
|  | Breaking Barriers: Providing Skin Cancer Education to the Homeless and Uninsured[10] |  | Not reported. |  | Not reported. | 2012-2015: inflammatory diseases (29%), benign growths (23%), and infectious diseases (14%) | Not reported. | Quality improvement project: 80% (n=57) surveyed patients never heard of BCC, majority did not use sunscreen, education intervention improving sun protection knowledge |
|  | Evaluation of biopsy management at student-run free clinic[11] |  | Not reported. |  | Not reported. | Not clearly discussed. Prominent biopsied lesions included: 6 basal cell carcinomas, 2 squamous cell carcinomas, 1 malignant melanoma, and 1 unusual melanocytic lesion | In 24 months, 54 patients underwent biopsy. Eleven punch biopsies and 43 shave biopsies were performed. | Not reported. |
|  | Analysis of cutaneous infections in homeless populations at student-run free clinic[12] |  | Not reported. |  | 100 patients diagnosed with conditions of infectious etiology (over 2 years, 2018-2020) | Tinea infection (23%), plantar warts (9%), folliculitis (9%), unspecified mycosis (6%), impetigo (5%), and scabies (5%). Treatment employed included liquid nitrogen cryotherapy (17%), topical antifungals (15%), triamcinolone cream (9%), doxycycline (8%), and clindamycin (8%) | Not reported. | Average time from symptom development to clinic 8.6 months, with median 2.5 months |
|  | 43071 Assessing the Impact of Volunteer Training at Dermatology Student-Run Free Clinic[13] |  | Not reported. | Not reported. | Not reported. | Not reported. | Not reported. | Survey of 29 preclinical demonstrations utility of education module in improving describing dermatology morphology, obtaining patient histories, and conveying findings to dermatology faculty |
|  | 43199 Analyzing Follow-Up Rates and Barriers to Care in Student-Run Free Clinic[14] |  | Not reported. | Not reported. | Not reported. | Not reported. | Not reported. | Survey of 49 patients found financial concerns and lack of insurance as significant barriers; 20/49 patients completed follow up appointments |
|  | 43091 Psychodermatologic Disorders in Patient Population at Student-Run Free Clinic[15] |  | Not reported. | Not reported. | Not reported. | August 2018 to January 2022 retrospective study of 158 psychodermatologic symptoms: 80% (n=127) psychodermatologic disorder (PD); main treatment: topical prescription (n=156) and 28% (n=44) as primary dermatologic with secondary psychiatric symptoms | Not reported. | Not reported. |
|  | 39999 Breaking Barriers in Underserved Communities and Improving Health Literacy Through a Student-Run Free Clinic[16] |  | Not reported. | Not reported. | Not reported. | Not reported. | Not reported. | QI project involving action plan for handouts with graphics, additional volunteer training, and renovations to clinic. 311 Patient surveys with improvement in comprehension of medical conditions (p=0.0165) and comprehension of treatment plans (0.0099) |
|  | Improving Medical Student Confidence Performing Skin Biopsies Through an Interactive Workshop[17] |  | Not reported. | Not reported. | Not reported. | Not reported. | Not reported. | Module on delivering biopsies resulted in statistically significant improvement via survey of student comfort and ability to perform punch and shave skin biopsies |
| **Cardinal Free Clinics: monthly Dermatology clinic** | Patient satisfaction in dermatologic care delivered by a medical–student-run free clinic[18] | Stanford Healthcare, community based-physicians & Stanford University student partnership, Stanford, CA | 62% patients of general clinic have annual income less than $25k, majority uninsured | 1x/month (Dermatology specialty days). Dermatology data for 2013-2015. General medicine clinic began 1990, 2nd general medicine clinic began 2005. | 71 (over 3 years) | Acne vulgaris (14%), Tinea (14%), Psoriasis (11%), Atopic Dermatitis (10%) | Biopsies  Weekend clinic providing general & specialty care was established first.  On-site Stanford-affiliated dermatologist & interpreted by Stanford dermatopathologists pro-bono | Philanthropy and the university contribute financially.  Stanford dermatopathology pro-bono |
| **CD Doydle Clinic (CDD)** | Establishing Dermatologic Care for the Homeless and Underserved at a Student-Run Clinic[19] | Dell Medical School, Austin, TX | 80% homeless (median age 49; race/ethnicity 53.3% white, 20% black, 26.7 % Hispanic) | 1x/month | 15 (4 dermatologic clinics over 4-month pilot) | Benign nevi | Skin care and screenings; social work; partial/full body skin exams; education and teach back; benign condition treatment in clinic; further work-up referred to local dermatologists  Local lidocaine, scalpels, curettes provided by residents' clinic; resident brought dermatoscopes; over-the-counter medications, materials, wound care supplies obtained via donations | Residents attendance = continuity of clinic; dermatologic screenings not previously conducted.  Barriers: resident/attending sign up cannot be scaled, having clinic supply dermatology supplies (liquid nitrogen, bx tools) |
| **UCSF Student-run clinic at the Multi Service Center (MSC)-South Homeless Shelter** | Survey of symptomatic dermatologic disease in homeless patients at a shelter-based clinic[20] | University of California (UCSF), San Francisco, CA | Homeless patients; age range: 19-78 (mean 48.7); 100 total patients (74 males, 25 females, 1 transgender); Race/ethnicity: 43 unknown, 26 white, 17 black, 2 Native American, 2 Asian, | 2x/month. Data from 2011-2015. | 136 dermatologic complaints | Inflammatory dermatoses (lichen simplex chronicus, psoriasis, atopic dermatitis), superficial fungal infections (tinea pedis, onychomycosis, cutaneous candidiasis), wounds and trauma (lacerations, surgical wounds, abrasions) | Due to limited resources, biopsies, KOH microscopy for tinea, bacterial and fungal culture, and other simple diagnostic tools were not available. Full-body exams also not possible in shelter setting.  Steroids (topical) 31 units, antifungals (topicals) 30 units, emollients 16 units | Visual diagnoses as barriers. No biopses, KOH microscopy, BCx, Fungal Cx, or other diagnostics; full-body skin exams no possible due to privacy, |
| **Paul Hom Asian Clinic (PHAC)** | Characteristics of patients seen at a dermatology free clinic, 2017-2020: a retrospective chart review[21] | University of California (UCD), Davis, CA | Age range: 12-78, mean 50; 48.6% Chinese, 29.4% other Asian, Non-Asian (22.0%); 52.3% uninsured; 47.7% insured | Data from 2017-2020 | 94 patients, 109 skin-related chief complaints (over 3 years) | Eczema, psoriasis, benign skin growths | Not reported. | Not reported. |
| **Pride Community Clinic (PCC)** | 41697 Predominant Dermatological Conditions in Female-to-Male Transgender Patients at Pride Community Clinic[22] | University of Texas Health Science Center at San Antonio, San Antonio, TX | LGBTQIA | Not reported. | September 2017 to June 2022: 15/54 with skin issues after starting HRT | Acne vulgaris (12/15), alopecia (2/15), skin lesion otherwise unspecified associated with undiagnosed GI disorder | Not reported. | Not reported. |
|  | 40673 Evaluation of a Monkeypox Educational Intervention in a LGBTQIA+ Student Run Free Clinic[23] |  | LGBTQIA | Not reported. | Not reported. | Not reported. | Not reported. | Educational module on monkeypox resulted in statistically significant improvement for 41 patients |
| **South Park Inn (SPI) Homeless shelter** | Dermatologic Conditions in a Shelter-Based Homeless Population: Lessons Learned from a Medical Student-Run Dermatology Clinic[24] | University of Connecticut Hartford, Connecticut | Median age (39), Mean age (37.2); ethnicity: 29.6% African American, 29.3% Hispanic, 25.3% Caucasian | Monthly | 275 (over 7.5 years, 2008-2016) | Acne vulgaris (1.7%), atopic dermatitis (10.6%), tinea pedis (9.2%); rate of skin malignancies (2.5%) | Biopsy and treatment, skin cancer prevention, screening  No procedural supplies (skin biopsy, excision, liquid nitrogen) available | Free medications/ supplies via donations/university funding (topical moisturizers, sunscreens, benzoyl peroxide wash, low-potency corticosteroids, antibiotics (clindamycin), antifungals (clotrimazole)  Majority patients lost to follow-up; low verification ability with histopathologic analysis; clinic not held in Summer  Attending and medical students: student writes chart notes under attending supervision |
| **Health Outreach Partnership of EVMS Students (HOPES)** | Addressing Dermatologic Health Disparities: Characterization of a Free Dermatology Clinic for an Uninsured Population[25] | Eastern Virginia School of Medicine, Norfolk, VA | 20 Spanish-speaking; 195 English-speaking; all patients uninsured; mean age 50 | Monthly, Data from 2012-2019 | 215 (over 7 years, 2012-2019). 8 patients/clinic day | Atopic dermatitis (n=26), seborrheic keratosis (n=21), acne (n=17); 23 skin cancer (13 BCC, 8 SCC, 1 melanoma, 1 NMSC) | 103 medications prescribed; steroids (55.3%), antibiotics (16.5%), antifungals (13.6%); Procedures: 27 biopsy, cryotherapy 24 patients, 14 steroid injections, 9 excisions, 5 KOH preparations | Follow up rate of 78%; Patients lack access to email/mobile devices  Two physicians (attending residents); two senior clinicians (senior medical student or PA); junior clinicians (junior medical students) |
| **HOYA Clinic** | Dermatologic Education in Under-Resourced Communities: A Collaboration With a Non-Profit and a Student-Run Free Health Clinic[26] | Georgetown University School of Medicine, Washington DC | Not reported. | Not reported. | Not reported. | Not reported. | Not reported. | Partnering with non-profit can allow dermatologic education and sharing of skin health (3059) products to SRC patients at 3 HOYA health fairs |
| **Referral from Squirrel Hill Health Center, federally funded community health center** | The Student Dermatology Clinic for the Underserved: A Service-Learning Model to Promote Skin Health Equity[27] | University of Pittsburgh Medical Center, University of Pittsburgh School of Medicine collaboration, Pittsburgh, PA | Not reported | Quarterly | 320 (over 8 years). 10 pts/week. | Psoriasis, verrucous carcinoma, lupus, vitiligo, HS, eczema, keratoacanthoma, lichen planus, sexually transmitted infections | Referrals to primary care, dermatology care clinics for continuity care | Biopsies read by University of Pittsburgh Physicians Dermatopathology Unit free of charge; DIG student coordinators oversee medical student volunteer and patient scheduling, while dermatology residents coordinate resident participation; all residents must participate in one service-learning activity annually  1 attending physician |
| **Community Health Advancement Program (CHAP)** | 24 Years of Student-Run Free Clinics: A Review of the Community Health Advancement Program (CHAP) Dermatology Clinic and Challenges Faced[28] | University of Washington School of Medicine + Downtown Emergency Service Center (shelter), Seattle, WA | Not reported | Weekly, began 1994 | Not reported | Scabies, lice, cellulitis, atopic dermatitis, tinea pedis, and diabetic foot ulcers | Not explicitly discussed (most treatment plans involve topical antibiotics/antifungals, oral antibiotics, and topical corticosteroids—all which CHAP stocks as high-yield medications) | CHAP has a paid staff program coordinator and faculty physician through UWSOM’s Department of Family Medicine.  Follow-up difficult due to spread of clinics and population served, past patient records are securely kept at the DESC facility with their nursing staff, and notes from previous patient visits seldom accessed at subsequent clinics  3-5 regular preceptors |
| **Urban student-run health clinic** | Dermatological Needs in an Urban Free Health Care Setting [29] | University of Alabama at Birmingham Heersink School of Medicine, Birmingham, AL | Median age 40, Ethnicities: White (26.2%), Hispanic/Latino (28.6%), Black 28.6% | Not reported | 57 patients (over 3 years, 2019-2021), 61 encounters. 4.1% of 1738 total patients with dermatologic complaints | 30 (49.2%) dermatitis, 12 (19.7%) growth/cysts, 7 (11.5%) acne | 73.7% (42) underwent treatment: 83.3% (35) medication, majority receiving topical therapy, 7 (16.7%) underwent procedural intervention (2 steroid injections, 2 punch biopsies, 1 excision biopsy, 1 lancing/drainage) | Board-certified dermatologists for dermatology  specialty days  Low follow-up (71.9%), though high patient adherence 81.2% among those able to follow up |
| **Student Family Healthcare Center (SFHCC)** | Assessing Skin Cancer Screening in a Student-Run Healthcare Clinic[30] | Rutgers New Jersey Medical School, Newark, NJ | 50 randomly selected charts reviewed, median age 46.76; 38% male, 62% female. 48% African American, 24% Hispanic, 28% other | Not reported | 50 charts reviewed | Not reported | Integrated skin cancer screenings only, within a general medicine clinic | 40 (80 percent) had been screened for skin cancer at some point during their time as a patient at the SFHCC, for a total of 110 times (average of 2.75 screenings per person) |
| **Unknown** | 42662 Dermatology for the Underserved at a Non-Profit Clinic in Charleston[31] | Charleston, North Carolina |  | March 2016 – May 2022. | 259 patients and 80 unique diagnoses | Not reported. | Not reported. | 19/53 patients seen by dermatologist within scope of PCP. Patients seen by dermatologist more likely to have premalignant skin condition diagnosis and receive indicated procedure |

1. Lin CP, Chow N, Rafael J, Bennett K, Tarbox M, Sturgeon A, et al. Dermatologic Care for the Uninsured West Texas Population at a Student-run Free Clinic. J Health Care Poor Underserved. 2021;32(3):1155-9. PMID: 34421019. doi: 10.1353/hpu.2021.0120.

2. Lin CP, Loy S, Boothe WD, Bennett K, Tarbox MB, Prabhu F, et al. Value of Dermatology Nights at a student-run free clinic. Proc (Bayl Univ Med Cent). 2020 Oct 26;34(2):260-1. PMID: 33678959. doi: 10.1080/08998280.2020.1834771.

3. Mirza FN, Valladares HC, Richards B, Suozzi KC. Meeting Dermatologic Needs in an Uninsured Population: Lessons Learned from a Referrals Cohort at a Student-Run Free Clinic. Yale J Biol Med. 2021 Sep;94(3):459-64. PMID: 34602883.

4. Linggonegoro D, Rrapi R, Ashrafzadeh S, McCormack L, Bartenstein D, Hazen TJ, et al. Continuing patient care to underserved communities and medical education during the COVID-19 pandemic through a teledermatology student-run clinic. Pediatr Dermatol. 2021 Jul;38(4):977-9. PMID: 34101255. doi: 10.1111/pde.14653.

5. Ramiro Rodriguez B, Orlando Martinez-Luna B, Rebecca Vasquez M. Delivering care for the underserved during COVID-19 through real-time teledermatology, a cross-sectional review of patients at a student-run free clinic in Dallas

. J Am Acad Dermatol. 2021;85(3):AB13.

6. Zhu C, Vu M, Nguyen T, Papanikolaou K, Browning J. Pattern of pediatric skin diseases at student-run free clinic

. J Am Acad Dermatol. 2022;87(3):AB190.

7. Zhu C, Bambekova P, Patel K, Patel RS, Browning JC. 13093 Retrospective review of skin cancer findings at student-run free clinic. Journal of the American Academy of Dermatology. 2020;83(6):AB5. doi: 10.1016/j.jaad.2020.06.107.

8. Patel R, Vu M, Wong J, Browning J. Travel burden for free dermatologic care in uninsured and homeless populations

. J Am Acad Dermatol. 2021 (3):AB75.

9. Papanikolaou K, Nguyen T, Zhu C, Vu M, Browning J. Predominant dermatologic issues in Hispanic patients at student-run free clinic

. J Am Acad Dermatol. 2022;87(3): AB193.

10. Altshuler J, Wallis L, Tracy A, Brown M, Siddiqui F, Nguyen MA, et al. Breaking Barriers: Providing Skin Cancer Education to the Homeless and Uninsured

. Free Research Collective. 2015;1:5-11.

11. Zhu C, Patel K, Guerrero G, Vu M, Wong J, Bambekova P, et al., editors. 24982 Evaluation of biopsy management at student-run free clinic

. American Academy of Dermatology; 2021: Journal of the American Academy of Dermatology.

12. Vu M, Wong J, Calderon F, Luu J, Schachter C, Browning J. Analysis of cutaneous infections in homeless populations at student-run free clinic. J Am Acad Dermatol. 2021;85(3):AB53.

13. Nguyen T, Shah A, Momin B, Papanikolaou K, Alfaro M, Bambekova P, et al. 43071 Assessing the Impact of Volunteer Training at Dermatology Student-Run Free Clinic. Journal of the American Academy of Dermatology. 2023;89(3):AB18. doi: 10.1016/j.jaad.2023.07.079.

14. Momin B, Nguyen TF, Shah A, Browning JC. 43199 Analyzing Follow-Up Rates and Barriers to Care in Student-Run Free Clinic. Journal of the American Academy of Dermatology. 2023;89(3):AB16. doi: 10.1016/j.jaad.2023.07.071.

15. Nguyen T, Papanikolaou K, Liu E, Momin B, Shah A, Alfaro M, et al. 43091 Psychodermatologic Disorders in Patient Population at Student-Run Free Clinic. Journal of the American Academy of Dermatology. 2023;89(3):AB79. doi: 10.1016/j.jaad.2023.07.321.

16. Zhu C, Browning J. 39999 Breaking Barriers in Underserved Communities and Improving Health Literacy Through a Student-Run Free Clinic. Journal of the American Academy of Dermatology. 2023;89(3):AB23. doi: 10.1016/j.jaad.2023.07.096.

17. Momin B, Shah A, Bambekova P, Browning J, Gilson R. Improving Medical Student Confidence Performing Skin Biopsies Through an Interactive Workshop. SKIN The Journal of Cutaneous Medicine. 2023:996–1000. doi: <https://doi.org/10.25251/skin.7.5.4>.

18. Pyles MN, Nkansah N, Sun BK. Patient satisfaction in dermatologic care delivered by a medical-student-run free clinic. J Am Acad Dermatol. 2016 Jun;74(6):1265-7. PMID: 27185434. doi: 10.1016/j.jaad.2015.12.036.

19. Teal L, Spitz K, Diven D. Establishing Dermatologic Care for the Homeless and Underserved at a Student-Run Clinic. J Stud Run Clin. 2020;6.

20. Contag C, Lowenstein SE, Jain S, Amerson EH. Survey of symptomatic dermatologic disease in

homeless patients at a shelter-based clinic. Our Dermatology Online. 2017. doi: 10.7241/ourd.20172.37.

21. Hai J, Nguyen M, Kim-Lim P, Wa Cheung K, Jan R, Tartar DM. Characteristics of patients seen at a dermatology free clinic, 2017-2020: a retrospective chart review. Dermatol Online J. 2021 Mar 15;27(3). PMID: 33865291.

22. Alfaro MA, Tran L, Quach E, Ren Y, Nguyen T, Garcia S. 41697 Predominant Dermatological Conditions in Female-to-Male Transgender Patients at Pride Community Clinic. Journal of the American Academy of Dermatology. 2023;89(3):AB76. doi: 10.1016/j.jaad.2023.07.307.

23. Alfaro M, Ayodele V, Park J, Quach E, Ren Y, Gravell C, et al. 40673 Evaluation of a Monkeypox Educational Intervention in a LGBTQIA+ Student Run Free Clinic. Journal of the American Academy of Dermatology. 2023;89(3):AB46. doi: 10.1016/j.jaad.2023.07.188.

24. Shahriari N, Torre K, Payette M, Murphy MJ. Dermatologic Conditions in a Shelter-Based Homeless Population: Lessons Learned from a Medical Student-Run Dermatology Clinic

. Connecticut Medicine 2017;81(6).

25. O'Connell KM, Bartholomew E, Villanueva AM. Addressing Dermatologic Health Disparities: Characterization of a Free Dermatology Clinic for an Uninsured Population

. Stud Run Clin. 2021;7.

26. Campbell JR, Imo BU, Barajas R, Lloyd HW, Moore E. Dermatologic Education in Under-Resourced Communities: A Collaboration With a Non-Profit and a Student-Run Free Health Clinic. J Drugs Dermatol. 2024 May 1;23(5):e128-e9. PMID: 38709688. doi: 10.36849/JDD.7820.

27. Patel BM, Humphrey V, J. James A. The Student Dermatology Clinic for the Underserved: A Service-Learning Model to Promote Skin Health Equity

. International Journal of Medical Students. 2022. doi: <https://doi.org/10.5195/ijms.2022.1086>.

28. Dhami A, Rennebohm M, Bienz D. 24 Years of Student-Run Free Clinics: A Review of the Community Health Advancement Program (CHAP) Dermatology Clinic and Challenges Faced

. J Stud Run Clin 2019;7.

29. Patel J, Kozar T, Sowell J, Chambers ME, Patel O, Mayo T. Dermatological Needs in an Urban Free Health Care Setting. Cureus. 2022 Nov;14(11):e31203. PMID: 36505149. doi: 10.7759/cureus.31203.

30. Wassef C, Keller SE. Assessing Skin Cancer Screening in a Student-Run Healthcare Clinic. 2013; Available from: <https://assets.bmctoday.net/practicaldermatology/pdfs/PD0613_CF_Screening.pdf>.

31. Barker C, Lateef A, Tharp P, Snyder A, Wallace L, Ramsetty A, et al. 42662 Dermatology for the Underserved at a Non-Profit Clinic in Charleston. Journal of the American Academy of Dermatology. 2023;89(3):AB146. doi: 10.1016/j.jaad.2023.07.585.
